# Design of a Wearable Eye-Movement Detection System Based on Electrooculography Signals and Its Experimental Validation

**DOI:** 10.3390/bios11090343

**Published:** 2021-09-17

**Authors:** Chin-Teng Lin, Wei-Ling Jiang, Sheng-Fu Chen, Kuan-Chih Huang, Lun-De Liao

**Affiliations:** 1 Australia Artificial Intelligence Institute, Faculty of Engineering and Information Technology, University of Technology, Sydney, NSW 2007, Australia; 2 Institute of Electrical Control Engineering, National Yang Ming Chiao Tung University, Hsinchu 30010, Taiwan; issacreal@gmail.com (W.-L.J.); kchuang.ece91g@g2.nctu.edu.tw (K.-C.H.); 3 Brain Research Center, National Yang Ming Chiao Tung University, Hsinchu 30010, Taiwan; 4 Institute of Biomedical Engineering and Nanomedicine, National Health Research Institutes, Zhunan Township, Miaoli City 35053, Taiwan; sanfo@nhri.edu.tw

**Keywords:** human–computer interface, electrooculography, eye-movement detection, fixation, saccade, blink

## Abstract

In the assistive research area, human–computer interface (HCI) technology is used to help people with disabilities by conveying their intentions and thoughts to the outside world. Many HCI systems based on eye movement have been proposed to assist people with disabilities. However, due to the complexity of the necessary algorithms and the difficulty of hardware implementation, there are few general-purpose designs that consider practicality and stability in real life. Therefore, to solve these limitations and problems, an HCI system based on electrooculography (EOG) is proposed in this study. The proposed classification algorithm provides eye-state detection, including the fixation, saccade, and blinking states. Moreover, this algorithm can distinguish among ten kinds of saccade movements (i.e., up, down, left, right, farther left, farther right, up-left, down-left, up-right, and down-right). In addition, we developed an HCI system based on an eye-movement classification algorithm. This system provides an eye-dialing interface that can be used to improve the lives of people with disabilities. The results illustrate the good performance of the proposed classification algorithm. Moreover, the EOG-based system, which can detect ten different eye-movement features, can be utilized in real-life applications.

## 1. Introduction

From the time computers were invented, the means of communication between humans and computers have evolved continuously. The human–computer interface (HCI) is a major research area in studying information transition methods. With the development of scientific and technological progress, computers are widely used in every area of human life. Additionally, people are increasingly relying on computers. This fact provokes the progression of the HCI research area. Some extraordinary HCI technologies have been proposed [1]. These include voice recognition [2,3], visual information [4], gesture control [5,6], methods based on brain signals, and infrared head-operated joysticks [7]. These methods were developed to provide a different way for people to use computers. Some of these methods were specifically designed for people with disabilities. People with severe diseases, such as amyotrophic lateral sclerosis (ALS), brainstem stroke, brain or spinal cord injury [8], cerebral palsy, muscular dystrophies, and multiple sclerosis, have difficulty conveying their intentions and communicating with other people in daily life [9,10,11].

With the development of HCI, methods have been developed to help these people communicate. Unlike traditional HCIs (a keyboard, a mouse, etc.), modern HCIs have played an important role in the area of rehabilitation. Among HCI systems based on biosignals, electrooculography-based systems are an important research area. There are several electrooculography (EOG)-based HCI applications for different purposes in the literature. The use of eye-based HCI systems to control computer functions provides an alternative way to communicate, e.g., via virtual keyboards [12,13], cursors [14] or even new interfaces [15], enabling assistive device control for people with disabilities via, e.g., prostheses [16] or powered wheelchairs [17]. Additionally, some applications focus on the user state, such as the analysis of the activity recognition of a person [18], analysis of reading activities [19] by wearing EOG goggles [20], or analysis of mental health [21]. The control of movable systems, such as robot arms [22] or entire robots [23,24], is also an emerging research area.

Currently, people with disabilities need many home care services [25,26]. For families with disabled members, how to help these families and offer them a better life is an important issue. The responsibility of looking after a patient may be taken by a housemaid or one of the family members, who has to quit his or her job as a result. Responsibility inevitably becomes an economic burden on the family and results in personal mental stress. Furthermore, it is a waste of human resources to society when productive people have to become full-time care workers. Thus, it is important to help people with disabilities and their families have better lives. In recent years, assistive technology for people with disabilities has gradually received more attention, and many methods have been proposed [27,28]. As computers become increasingly available in our daily lives, new research in the area of HCIs [29] arises that focuses on the commutation between humans and machines. EOG signals can be used to carry out communication between users and computers. Because of the natural characteristics of EOG signals, eye movements contain abundant information that corresponds to the users’ mental condition, health state, and more. Thus, a user’s state can be understood by analyzing the user’s eye movement and eye position. The primary application of eye tracking is to study human vision; eye tracking is also applied to medical diagnosis. To assess eye movement, a few methods have been proposed (i.e., video-based methods and infrared reflection methods).

In fact, the measurement method and wireless limitations have restricted the range of HCI experiments and the corresponding applications. To improve these limitations of eye measurement, different kinds of EOG signal measurements have been developed. Some of these are measured by infrared oculography. Infrared oculography methods are based on the reflection of near-infrared light on the cornea. Infrared oculography has advantages in scholarly studies because it can be used in the dark and offers a high spatial resolution. However, some drawbacks of using infrared oculography exist, such as the high cost of the manufacturing procedure of infrared sensors and the discomfort caused by their rigid substrate. In addition, eye damage is caused by long-term infrared oculography experiments. Some of these are measured by a video-based eye tracker. This method uses a camera to capture the image information of the eyes. In addition, the eye information can be computed for the eye position via an image processing algorithm. Thus, many algorithms have been proposed to improve image processing, such as video systems [30,31], dual-Purkinje images [32], and optical-type eye-tracking systems [33]. However, the disadvantages of this measurement process are the restriction of user movement and discomfort experienced in long-term experiments. Some of these factors are measured by a scleral search coil. Several induction coils wrapped in silicone are made in the soft lenses, and eye movement changes the magnetic field, which can result in the detection of eye movement. However, this method has the risk of visual impairment and uses an invasive method to measure eye movements, which may cause damage to the eyes.

In this study, a wearable EOG-based HCI system for ten-directional eye-movement detection consisting of dry sensors/electrodes, a wireless acquisition device, and an eye-movement classification method is proposed to classify the EOG signals. Dry electrodes can be utilized with good signal quality and can provide good electrical conductivity, resulting in the effective acquisition of EOG signals. In contrast to other HCI systems, users can reduce the skin preparation processes using dry electrodes and achieve highly accurate EOG signals. Therefore, the developed wireless HCI system has the potential to be used in daily life applications in the future.

## 2. Materials and Methods

### 2.1. System Overview

**Figure 1:**
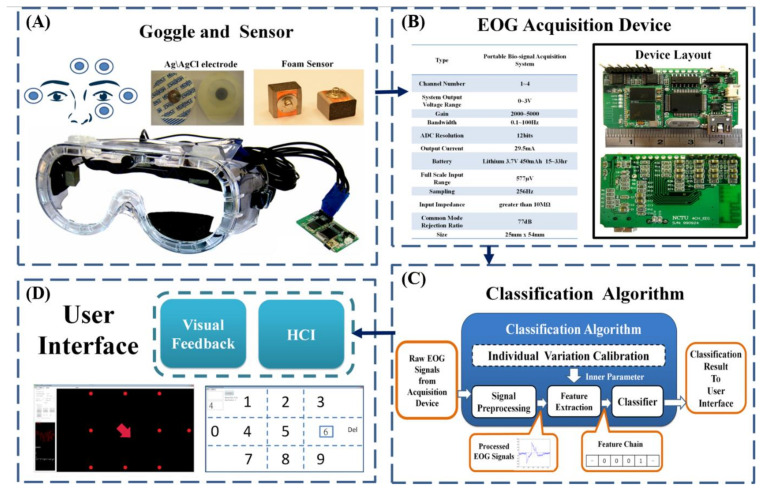
System overview of the proposed eye-movement detection system. (**A**) The EOG signals are collected from five locations on the face. The electrical potentials are detected through developed dry sensors. (**B**) Specifications of the developed EOG signal acquisition device and its layout. After the EOG signals are acquired, these tiny signals are processed and transmitted to the operation platform. (**C**) Diagram of the classification algorithm used. We chose a personal computer (PC) as our operation platform in this study. The PC unit performs the major task of HMI system computation. In the PC software, the eye-movement detection and eye-movement classification algorithms are executed. (**D**) The user interface developed in this study. The classification result is presented on a monitor via the user interface. Additionally, we design an eye-dialing interface in our system.

In this study, our goal is to build a sophisticated eye-movement detection system. The proposed system can classify what types of eye movement occur when people use their eyes in their daily lives, as shown in Figure 1. Furthermore, we extend the application of this system to the HCI level to enable people with disabilities to live better lives. Our system is composed of two parts. The hardware part comprises the EOG signal acquisition device and the form sensor [34] (Figure 1A,B). The other part is the classification algorithm (Figure 1C). The software algorithm was built within the operation platform (Figure 1C,D). Due to the characteristics of our algorithm, the system does not require much computation to conduct classification. Thus, the choice of operation platform can vary, such as a personal computer (PC), smartphone or tablet. In this study, we chose a common PC as our operation platform. Our system works as follows. First, the EOG signal is collected from the user’s face (Figure 1A). Electrical potentials are detected through sensors. After the EOG signals are acquired, these tiny signals are processed and transmitted to the operation platform (Figure 1B–D). The PC unit performs the major task of human-machine interaction (HMI) system computation. In the PC software, eye-movement detection and eye-movement classification algorithms are executed. The classification result is then presented for monitoring. Additionally, we design an eye-dial interface for our system.

### 2.2. The EOG Signal Acquisition Device

**Figure 2:**
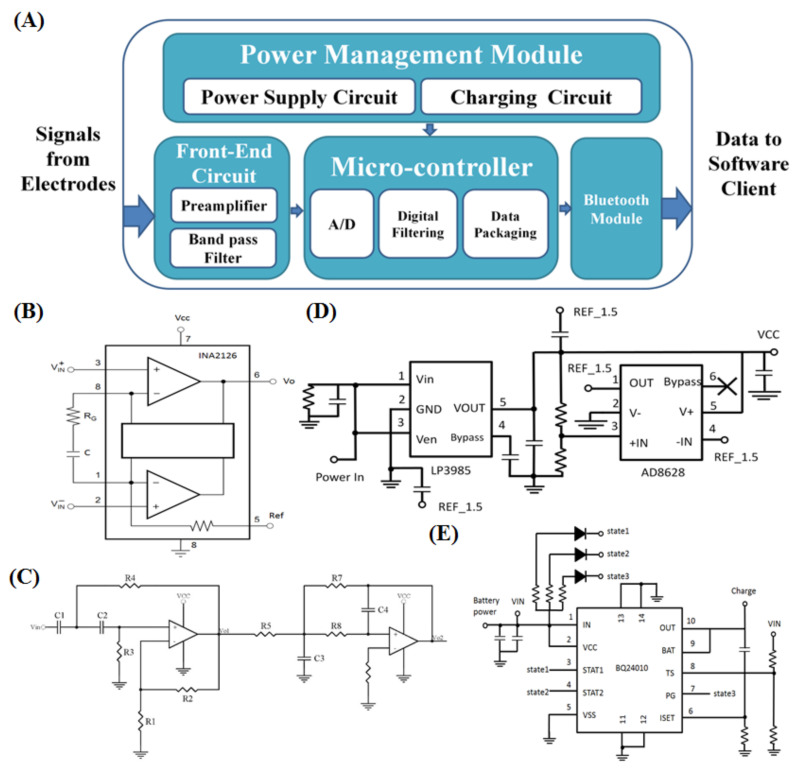
(**A**) Diagram of the portable biosignal acquisition unit. The portable biosignal acquisition unit can be divided into four parts: (1) front-end filter circuit, (2) microcontroller unit, (3) power management circuit and (4) wireless transmission. (**B**) Circuit design of the preamplifier. (**C**) Circuit design of the bandpass filter. (**D**) Circuit design of the power supply circuit. The maximum output current can support 550 mA. The turn-on time can reach 200 µs. We also use a voltage divider circuit to divide the VCC voltage to generate a virtual ground voltage and use a unity amplifier constructed from AD8628 as a voltage buffer. (**E**) Circuit design of the charging circuit. For portability, we use a Li-ion battery pack as a power supply source in our biosignal acquisition system. Additionally, to make the best use of the benefits of the battery, we add a charging circuit to the system. As the core of the charging circuit, we use BQ24010DRC, which is a highly integrated Li-ion and Li-pol linear charge management device. It offers an integrated power FET and current sensor, reverse blocking protection, high-accuracy current and voltage regulation, charge status, and charge termination in a small package. When the battery voltage falls below a preconfigured threshold, the charging circuit detects and begins to charge. The charging circuit also provides a protective mechanism to avoid overcharging or overdriving.

To develop a sophisticated HCI system, a well-performing biosignal measurement apparatus is necessary. The portable EOG signal acquisition device can be divided into four parts: (1) front-end filter circuit, (2) microcontroller unit, (3) power management circuit, and (4) wireless transmission. It is a lightweight, wireless recorder of physiological signals. A diagram of the portable biosignal acquisition unit is shown in Figure 1B and Figure 2A.

#### 2.2.1. Front-End Filter Circuit

The front-end circuit consists of a preamplifier and a bandpass filter. In some research, circuit designers preferred to use unit-gain filters and one variable-gain amplifier. Moreover, they did not use a high-pass filter to cut off the noise in the low-frequency band. We designed a three-stage high-pass filter and a two-stage low-pass filter to make the EOG signal clear to fit our goal.

#### 2.2.2. Preamplifier

In the first stage, we use the instrumental amplifier INA2126 to preprocess the tiny biosignals. From the point of view of biosignal circuit design, power consumption and noise degeneration are two important factors. INA2126 has a very low quiescent current, which reduces energy dissipation. Minimizing the amount of power wasted is important when designing a portable acquisition system that has only a battery as its power supply. Otherwise, INA2126 has a high common-mode rejection ratio (CMRR) of approximately 90 dB.

A high CMRR is important in applications. An amplifier with a high CMRR can suppress unwanted signals to be collected in the inputs. We use the instrumental amplifier INA2126 not only as a gain amplifier but also as a one-stage high-pass filter by adding a capacitor. The output voltage of INA2126 corresponds to the voltage on the reference terminal. The preamplifier circuit design is shown in Figure 2B.

#### 2.2.3. High-Pass Filter

In this study, operational amplifiers were used to achieve the function of a high-pass filter, as they are suitable for amplifying low-frequency signals. The AD8609 is a quad micropower rail-to-rail input and output amplifier; with the selection of a low dc offset, it serves as the bandpass filter. Figure 2C shows high-pass filter and low-pass filter circuits. The 3 dB cutoff frequency of the high-pass filters is determined by passive components R3, R4, C1, and C2. Passive components R7, R8, C3, and C4 determine the 3 dB cutoff frequency of the low-pass filter. A circuit comprising these bandpass filters and an amplifier is designed, as shown in Figure 2C, the gain of which is determined by passive components R1, R2, R5, and R7.

#### 2.2.4. Microcontroller Unit and Firmware Design

For the data acquisition system, a controller is needed to organize the analog-to-digital converter (ADC) operation and encode the digital data to the Bluetooth module by the UART port. When the preprocessing of biosignals is completed, we need a control unit to digitalize and convey the data to the PC unit. Considering the power and weight limitations, the MSP430 is well suited for wireless radio frequency (RF) or battery-powered applications. The MSP430 incorporates a 16-bit reduced instruction set computer (RISC) CPU, peripherals, and a flexible clock system that interconnect using a von Neumann common memory address bus (MAB) and memory data bus (MDB).

In these data acquisition systems, we use MSP430 F1611 as the main controller. The flash memory for firmware is 48 KB plus 256 B and 10 KB RAM for running the program. We use a built-in 16-bit timer, a fast 12-bit A/D converter, one universal serial synchronous/asynchronous communication interface (USART) and a 4 MHz external oscillator to develop our design [35]. In the next few sections, we briefly explain the functions of each.

#### 2.2.5. Timer Interrupt

The interrupt function is utilized to set the ADC sampling rate. The configuration of MSP430F1611 is based on the inner timer/counter register. Editing the value in the timer registers, users can operate the timer in several modes. In our design, we make the timer operate in “up mode”. In this mode, the timer repeatedly counts up to the value of register TACCR0. When the timer value equals TACCR0, the timer restarts, counting again from zero.

In our system, we use a 4-MHz crystal oscillator as the system clock source. Consequently, if the sampling rate of the system is set to 256 Hz, TACCR0 must be set to 15,625 Equation (1).

(1) $$\mathrm{TACCR}0=\frac{4M\;\mathrm{Hz}}{256\;\mathrm{Hz}}=15,625$$

#### 2.2.6. Analog-to-Digital Converter

When biological information is collected from the front circuit, we need to transform it into a digital signal. In our design, we choose the built-in ADC to address this task. The ADC12 module supports fast, 12-bit analog-to-digital conversions. The module implements a 12-bit successive approximation register (SAR) core, sample selection control, reference generator and a 16-word conversion-and-control buffer. The conversion-and-control buffer allows up to 16 independent ADC samples to be converted and stored without any CPU intervention.

The continuous signals can be input into the ADC module with port P6 (A0-A7) pins. The input multiplexer is a break-before-make type to reduce input-to-input noise injection resulting from channel switching. The ADC12 module is configured by three control registers: ADC12CTL0, ADC12CTL1 and ADC12MCLTx. The MSP430 ADC function has two operation modes. One is the extended sample mode, and the other is the pulse sample mode, which is used in this paper. The analog-to-digital conversion process is as follows. First, conversion is initiated with a rising edge of the sample input signal (SHI). The SHI is set by an interrupt vector routine at the desired sampling rate. In our system, when the ADC is activated by the timer interruption, the signal of each channel is sampled and converted into digital data. Each conversion requires 13 ADC12CLK cycles, including conversion and storage of the result in the ADC12MEMx conversion memory registers.

The ADC result of each channel will be 12 bits long in the form of an unsigned integer. The core uses two programmable/selectable voltage levels (VR+ and VR−) to define the upper and lower limits of the conversion. The input channel and the reference voltage levels (VR+ and VR−) are defined in the conversion-control memory. The conversion formula for the ADC result is Equation (2):

(2) $$\mathrm{Digital}\;\mathrm{value}=4095\times \frac{V_{\mathrm{in}}-V_{-}}{V_{+}-V_{-}}$$

When the conversion results are written to a selected ADC12MEMx, the corresponding flag in the ADC12IFGx register is set. Upon detection of the target flag being set, an ADC interrupt service routine is started. In this service routine, we utilize a digital filter to reduce noise, and then filtered data are encoded in some specific ordering before the data are sent to the Bluetooth module.

#### 2.2.7. Moving Average

Despite noise filtering at the hardware level, some high-frequency noise could still corrupt our signal in an unexpected way. Thus, to solve this problem, we need to introduce a filtering process at the firmware level. In digital signal processing, many mature methods exist. To meet the hardware limitations and our needs, we utilize the moving average method. The moving average, also called the rolling average, is the basic type of finite impulse response (FIR) filter in the digital signal processor (DSP) domain. The moving average is most commonly used with time-series data to smooth out short-term fluctuations and highlight longer-term trends or cycles. The choice between short-term and long-term and the setting of the moving average parameters depend on the application requirements. Mathematically, the moving average is a type of convolution and is similar to the low-pass filter used in signal processing. The moving average filter is optimal for a common task: reducing random noise while retaining a sharp step response. This makes it the premier filter for time-domain encoded signals. Now, consider an M-point sequence *x*[*n*]; we want to transform this sequence into a new sequence *y*[*n*] through an N-point moving average. This means that each element of the output *y*[*n*] is the average of N values in the order of the input sequence *x*[*n*].

Its input–output relation can be presented as the following difference equation Equation (3):

(3) $$\begin{array}{lll} y\left[n\right] & = & \frac{1}{N}\left(x\left[n\right]+x\left[n+1\right]+\cdots +x\left[n+N-1\right]\right)\\ & = & \frac{1}{N}\sum_{k=0}^{N-1}x\left[n+k\right] \end{array}$$

As mentioned above, the recorded signals are easily interfered with by 60-Hz noise, especially when the acquisition circuit is located near electric appliances. The original sine wave was contaminated by 60-Hz power-line noise. After filtering by using a moving average with a 5-point moving window, we found that the moving average could effectively remove the power-line noise [35].

Given a continuous noise signal x(t) with frequency F Hz, it is apparent that the integral within 1/F sec is equal to zero Equation (4).

(4) ∫01Xxt=0

We now consider the digital situation. The above equation can be extended to a digital form Equation (5). This means that the summation of all discrete signals with one period is equal to zero.

(5) $$\overset{\mathrm{All}\;\mathrm{signals}\;\mathrm{with}\;\mathrm{one}\;\mathrm{period}}{\underset{k=0}{\sum }}X\left[n+k\right]=0$$

Hence, we can use the moving average method to remove unwanted noise. The moving window size is determined by both the sampling rate and the noise frequency. When the noise signal is digitalized at a sampling rate S, we know that the number of sampled noise signals within one period is S/F. Thus, we obtain the following equation Equation (6):

(6) $$\mathrm{Moving}\;\mathrm{window}\;\mathrm{size}=\frac{\mathrm{Sampling}\;\mathrm{rate}\;S}{\mathrm{noise}\;\mathrm{frequency}\;F}$$

#### 2.2.8. UART Interface

Once the data are encoded, they are restored to the data buffer. Once the data buffer is full, we send the data to the Bluetooth module. MSP430 F1611 provides 3 types of data transmission protocols. We choose the UART interface as our approach to making use of the Bluetooth module.

In asynchronous mode, the USART connects the MSP430 to an external system via two external pins, URXD and UTXD. To initiate the UART transmit mode, we need to properly configure the operation settings, such as the character length, number of stop bits, and, most importantly, the transmitting baud rate.

The USART baud rate generator is capable of producing standard baud rates from nonstandard source frequencies. The baud rate is generated by dividing the BRCLK that could be sourced from SMCLK, ACLK or UCLKI. The maximum USART baud rate is one-third of the UART BRCLK source clock frequency. For a given BRCLK clock source, the baud rate used determines the required division factor N Equation (7):

(7) $$N=\frac{\mathrm{BRCLK}}{\mathrm{baud}\;\mathrm{rate}}$$

The division factor N is often a noninteger value, for which the integer portion can be realized by the prescaler/divider. In our system, BRCLK is 4 MHz, and the baud rate is 115,200 bit/s. After the initialization of the UART, the microcontroller could transmit the filtered data by moving the average to the Bluetooth module via the UART.

#### 2.2.9. Power Management

In our system, the power management circuit consists of two parts: a power supply circuit and a charging circuit.

#### 2.2.10. Power Supply Circuit

According to the integrated circuit (IC) working voltage and the analog circuit design, we set the operating voltage VCC at 3 V and the virtual ground of the analog circuit at 1.5 V. To provide a stable operating voltage, an LP3985 regulator is used to regulate the battery voltage to 3 V. LP3985 is a micropower, 150 mA, low noise, and ultralow dropout complementary metal-oxide semiconductor (CMOS) voltage regulator. The maximum output current can support 550 mA. Furthermore, the turn-on time can reach 200 µs. We also use a voltage divider circuit to divide the VCC voltage to generate the virtual ground voltage and utilize a unity amplifier constructed from the AD8628 to provide a voltage buffer. The entire power supply circuit is shown in Figure 2D.

#### 2.2.11. Charging Circuit

We use a Li-ion battery pack as a power supply source in our biosignal acquisition system for portability. Additionally, to make the best use of the benefits of the battery, we add a charging circuit to the system. The charging circuit is shown in Figure 2E. In the charging circuit, we use BQ24010DRC, which is a highly integrated Li-ion and Li-Pol linear charge management device, as the core of the charging circuit. It offers integrated power field-effect transistors (FETs) and current sensors, reverse blocking protection, highly accurate current and voltage regulation, charge status, and charge termination in a small package. When the battery voltage falls below a preconfigured threshold, the charging circuit will detect this event and begin to charge. The charging circuit also provides a protective mechanism to avoid overcharging or overdriving. Based on the design, the system can be fully operated for approximately 33 h once the battery is fully charged.

#### 2.2.12. Wireless Transmission

Bluetooth is a wireless protocol utilizing short-range communication technology to facilitate data transmission over short distances from fixed and/or mobile devices. The intent behind the development of Bluetooth was to create a single digital wireless protocol capable of connecting multiple devices and overcoming issues arising from the synchronization of these devices.

In this study, Bluetooth module BM0203 was used. BM0203 is an integrated Bluetooth module used to ease the design gap; it contains Cambridge Silicon Radio (CSR) BlueCore4-External as the major Bluetooth chip. CSR BlueCore4-External is a single-chip radio and baseband IC for Bluetooth 2.4-GHz systems including enhanced data rates (EDRs) up to 3 Mbps. It interfaces with 8 Mbit of external flash memory. When used with the CSR Bluetooth software stack, it provides a fully compliant Bluetooth system to v2.0 of the specification for data and voice communications. All hardware and device firmware of BM0203 are fully compliant with the Bluetooth v2.0 + EDR specification. Bluetooth operates in the high-frequency band to transmit wireless data; thus, it can be completely employed by using a printed circuit board (PCB) antenna. After we design the proposed acquisition circuit, we implement it on the PCB board. The device specifications and PCB layout are shown in Figure 1B.

### 2.3. Software Algorithm Design

In this section, we turn our attention to the development of a software algorithm. The algorithm can be divided into four major functions: (1) individual parameter calibration, (2) signal preprocessing, (3) feature extraction, and (4) classification. The user interface can output the real-time results of high-accuracy eye-movement detection. Furthermore, we propose an application based on eye movement to help persons with disabilities. In Section 2.3.1, we explain the target movement types and the reason why we chose these types. In Section 2.3.2, the variation problem is discussed. Moreover, we present the electrode placement of our system. Finally, the classification algorithm is introduced. We explain how each function of our system works at length.

#### 2.3.1. Target Types of Eye-Movement Detection

**Figure 3:**
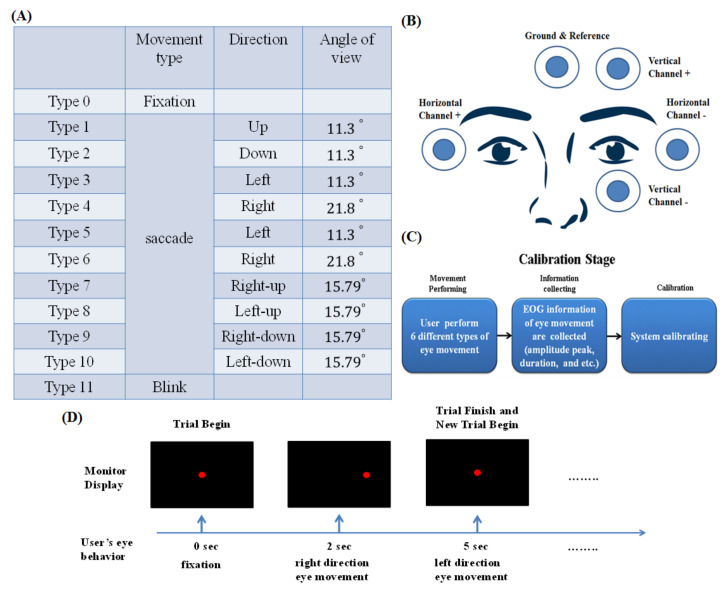
(**A**) Details of the target detection types, including the eye-movement type, eye-movement direction and moving angle. Additionally, for convenience in the subsequent study and experiments, we give each target movement a type number. (**B**) Electrode placement. (**C**) Calibration procedure. (**D**) Time sequence of the type 1 trial in the calibration stage.

As mentioned before, there are three main basic types of eye movements: saccade, smooth pursuit and vergence. In this study, we mainly focus on saccade movements. This is not only because the EOG signals corresponding to saccades are much easier to analyze but also because of their importance and frequent occurrence in daily life. Moreover, although blinking and fixation are not types of eye movement, we take these two eye states into consideration when developing the eye-movement detection system. The target types that the system will detect include 10 saccade movement types, the fixation state and blink. Regarding the saccade movement types, there are saccades in the up, down, left, right and other oblique directions. Details of the target detection types are listed in Figure 3A, including the eye-movement type, direction of eye movement and moving angle. Additionally, we give each target movement a type number for convenience to facilitate the following study and experiments.

#### 2.3.2. Variation Problem and Electrode Placement

In biosignal measurements, these signals are rarely deterministic. The magnitude of a biosignal varies with time even when we control all variables as much as possible. In this paper, the EOG magnitude is an important data feature utilized in the analysis. Thus, the variation problem becomes a neglected issue. When utilizing EOG as an eye-tracking tool, many factors associated with EOG variation should be carefully controlled. These factors are perturbations caused by other biopotentials, such as electroencephalography (EEG) and electromyography (EMG), in turn, brought about by the acquisition system, plus those due to the positioning of the electrodes, skin-electrode contacts, head and facial movements, lighting conditions, blinking, etc. Among the above factors, the positioning of electrodes and skin-electrode contacts plays an important role in determining signal quality. Thus, we surveyed many proposed EOG papers, and from them, we chose the electrode placement, as shown in Figure 3B. We place two electrodes on the temples of a person to acquire horizontal EOG signals. Additionally, we placed the ground terminal electrode between the eyebrows. Finally, because the two eyes of a person seldom move independently, we place a pair of electrodes under and beneath only the left eye on the face, as depicted in Figure 3B.

#### 2.3.3. Algorithm

The main data analysis method is built within the operation platform. This algorithm is composed of four parts: (1) individual parameter calibration, (2) signal preprocessing, (3) feature extraction and (4) classification.

##### Individual Parameter Calibration

For each user, before their use of this system, the system executes a pretest session, as shown in Figure 3C,D. In this session, the system collects eye-movement information from each user (Figure 3C). This information will facilitate system operation in the future. This function is performed to solve the variation problem. The variation sourced from the factors of one person is called intrasubject variation. In addition, because the habits of eye usage are different, another variation between each person, called intersubject variation, is generated. EOG intersubject and intrasubject variations always occur in our study regardless of dedicated efforts to prevent it. The intersubject variation will corrupt our analysis result. Hence, we design a calibration function to fix the intersubject variation problem (Figure 3C,D). When a user is new to this system, the system operates in the calibration stage to solve the intersubject and intrasubject variation problems. The calibration stage is composed of 6 trials, each of which is executed two times. The system proposes that users perform 6 types of eye movements in sequence. We now illustrate the pretest type 1 trial as an example. It proceeds as follows: There is a red dot in the middle of the LCD screen, at which users must stare. After a few seconds, the red point moves right for a quarter of the screen length. The red point remains at the new position temporarily. After a few seconds, the red point returns to the initial position at the center of the screen. Each type of movement is required to be carried out two times.

**Figure 4:**
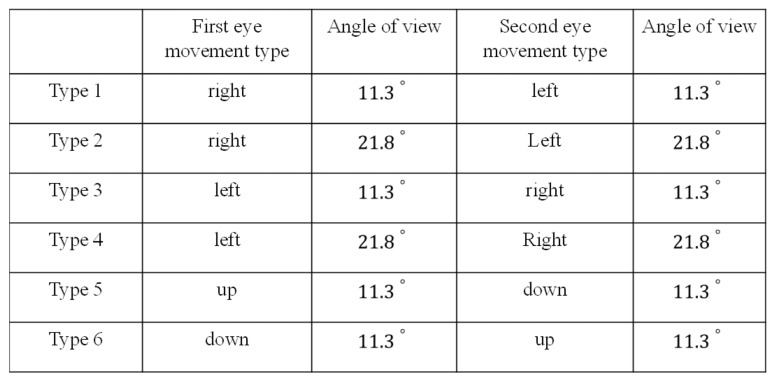
Details of the eye movements performed in the calibration stage.

As explained above, each type of trial involves two eye movements. In a type 1 movement, the subject first executes a right-direction eye movement, and then a left-direction eye movement is executed, with users returning their gaze to the initial position. Each eye-movement type contains different movement conditions. Details are listed in Figure 4. After the 6 types of movements are executed, the system algorithm computes the signal characteristics. These include the mean value and standard deviation values of the positive peak value, negative peak value, rise time and high-level time. The system then passes these arguments to the feature extraction function.

##### The Signal Preprocessing

In this stage, first, we group 4 channels into two pairs according to their source type. One is vertical EOG, and the other is horizontal EOG. We divide the channels into two pairs, for example, CH1-CH2 and CH3-CH4. This procedure is very important. When people perform eye movements, EMG always occurs. These EMG signals are contained in the EOG result when we acquire signals from a subject’s face. These EMG signals can be collected from everywhere on the face. They resemble a kind of common-mode noise.

Given noise n(t) originating primarily from the EMG and true EOG signals $E_{1}\left(t\right)$ and $E_{2}\left(t\right)$, the signals collected from our acquisition system are $X_{1}\left(t\right)$ and $X_{2}\left(t\right)$. We can express $X_{1}\left(t\right)$ and $X_{2}\left(t\right)$ in the following form (Equations (8) and (9)):

(8) $$X_{1}\left(t\right)=E_{1}\left(t\right)+n\left(t\right)$$

(9) $$X_{2}\left(t\right)=E_{2}\left(t\right)+n\left(t\right)\;$$

Thus, we can subtract the two signals to remove noise n(t) as follows (Equations (10) and (11)):

(10) $$X_{1}\left(t\right)-X_{2}\left(t\right)=E_{1}\left(t\right)+n\left(t\right)-\left(E_{2}\left(t\right)+n\left(t\right)\right)$$

(11) $$=E_{1}\left(t\right)-E_{2}\left(t\right)$$

**Figure 5:**
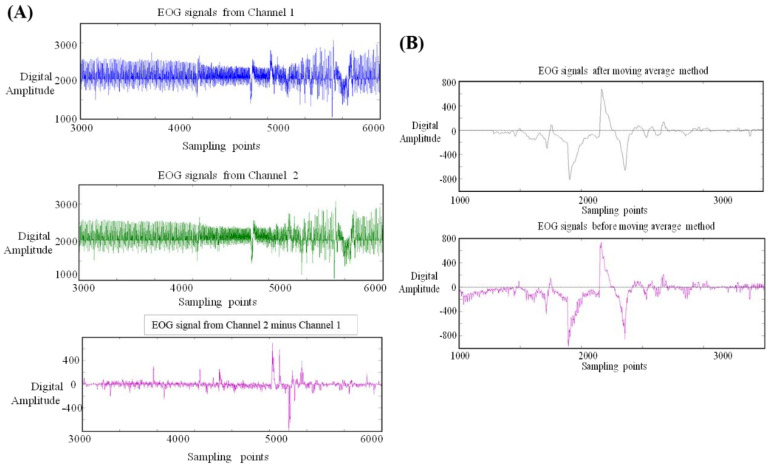
(**A**) Removal of common-mode noise. There are three pictures of EOG signals, from which we can clearly observe the removal of common-mode noise. Although the signal is filtered in the data acquisition device, there sometimes still exists some unexpected interference. In addition, the raw data transmitted to the operation platform always have errors that may be sourced from noise or from real biosignals. These error components will negatively impact our classification result. As a consequence, we reapply the moving average method to remove unwanted signal errors and smooth out the signals. In the preprocessing stage of the algorithm of the operation platform, we use a 15-point window size (**B**). (**B**) Difference between processed and original EOG signals. We can see the difference between the original signals and filtered signals. The signals after applying the moving average method are clearly smooth. This is in contrast to our feature extraction method.

In Figure 5A, there are three pictures of EOG signals; as shown, common-mode noise is removed. Although the signal is filtered in the data acquisition device, there often exists some unexpected interference. In addition, the raw data transmitted to the operation platform always have errors that may originate from noise or from the use of real biosignals. These error components negatively impact our classification result. As a consequence, we apply the moving average method again to remove any unwanted signal errors and smooth signals. In the preprocessing stage of the algorithm of the operation platform, we use a 15-point window.

As shown in Figure 5B, we can see the difference between the original signals and filtered signals (Figure 5B). The signals after the application of the moving average method are obviously smooth. Although the original characteristic of each signal could also be distorted in this process, this distortion does not impact our classification. This is in relation to our feature extraction method, which is explained in the next section.

##### Feature Extraction

**Figure 6:**
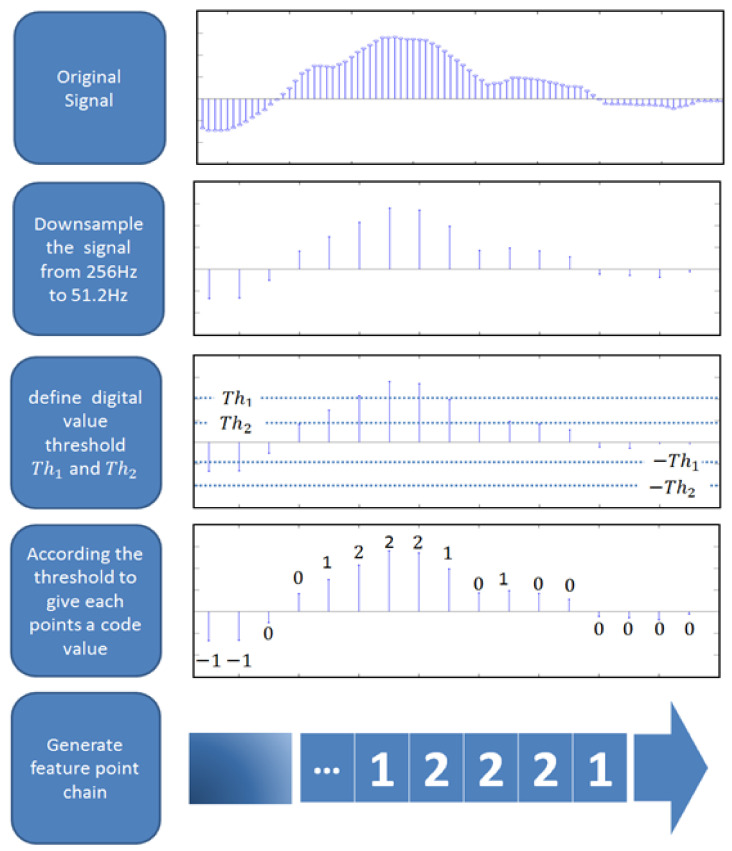
Feature extraction of the ECG signals. First, to reduce the computation amount, we downsample our data from 256 Hz to 51.2 Then, we encode each data point with a value ranging from −2 to +2. The values are given according to the digital amplitude value of the points. Similar to what we discussed in the previous sections, we acquire some eye-movement information in the calibration stage. Through these calibration arguments and the standard database, we define two voltage thresholds: ${\mathrm{Th}}_{1}\;$ and ${\mathrm{Th}}_{2}$. With these two values, we give each data point a coding value according to Equation (1). Once the coding values are determined, these values are formed into a sequence.

After preprocessing, we extract the data features in this stage to be used by the classifier. The steps are as follows, as shown in Figure 6. First, to reduce the computation amount, we downsample our data from 256 Hz to 51.2 Hz. Then, we encode each data point with a value. This value ranges from negative two to positive two. The values are given depending on the digital amplitude value of the points. Similar to what we discussed in the previous sections, in the calibration stage, we acquired some eye-movement information. Through these calibration arguments and the standard database, we define two voltage thresholds: ${\mathrm{Th}}_{1}$ and ${\mathrm{Th}}_{2}$. With these two values, we give each data point a coding value as follows Equation (12):

(12) fx=−2,       |          x≤−Th2−1,   −Th2<x≤Th10,  | −Th1<x≤Th1 1,    Th1<x≤Th22,           Th2<x .

Once the coding values are determined, these values form a sequence. In this study, we call this sequence the feature chain, which is used in the classification stage.

##### Classification

**Figure 7:**
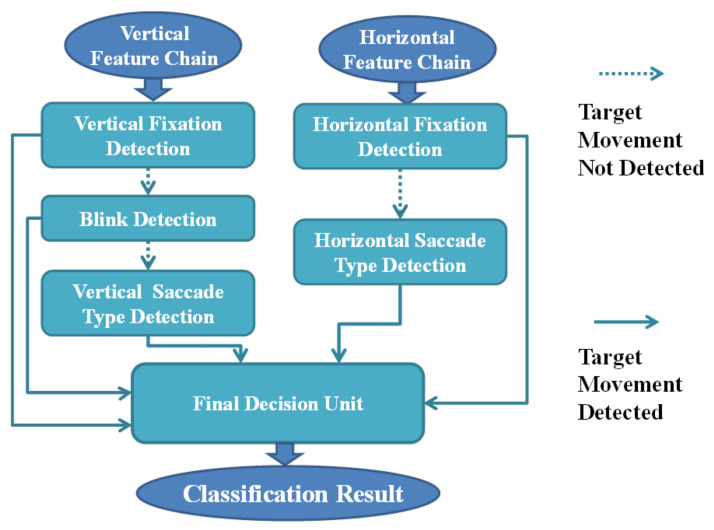
Detailed structure of the classifier. First, vertical feature chain information is transmitted to the fixation detection unit to determine whether there is any movement in the vertical direction of the user. Once movement does occur, the system passes the vertical feature chain to the blink detection unit. In the same procedure, if the detection unit recognizes the blinking motion input data, the unit outputs an inner parameter to the final decision unit. Otherwise, the input data are again passed to the next unit, i.e., the vertical movement detection unit. This unit may play the most important role in the whole vertical detection branch. In this unit, the system recognizes what type of saccade eye movement happens and transmits the classification parameter to the final decision unit. In the horizontal detection branch, the system process flow is similar to that of the vertical branch except for not having a blink detection unit. After the final decision unit collects all inner classification parameters, it integrates all this information and computes the final classification result.

After the feature chains are sent to the classifier, the vertical feature chain and horizontal feature chain are separately processed in different detection branches (Figure 7). Each detection process can be further divided into subdetection units. In these subdetection units, the system searches the database to match the target eye-movement pattern from the feature chain. If the subdetection units recognize the target pattern existing in the feature chain, it outputs an inner parameter to the final classification unit. If no pattern matching occurs in the current detection unit, the current unit outputs null to the final decision unit and passes the feature chain to the next unit. As depicted in Figure 7, first, vertical feature chain information is transmitted to the fixation detection unit to determine whether there is any movement in the vertical direction of the user. Once movement does exist, the system passes the vertical feature chain to the eye-blink detection unit. In the same procedure, if the detection unit recognizes the blinking action in the input data, the unit outputs an inner parameter to the final decision unit. Otherwise, the input data are again passed to the next unit, i.e., the vertical movement detection unit. This unit may play the most important role in the total vertical detection branch. In this unit, the system recognizes what type of saccade eye movement occurs and transmits the classification parameter to the final decision unit. In the horizontal detection branch, the system process flow is similar to that of the vertical branch except for not having a blink detection unit. After the final decision unit collects all inner classification parameters, it integrates all this information and computes the final classification result. The result is shown on the screen as a form of visual feedback.

##### Details of the Detection Units

**Figure 8:**
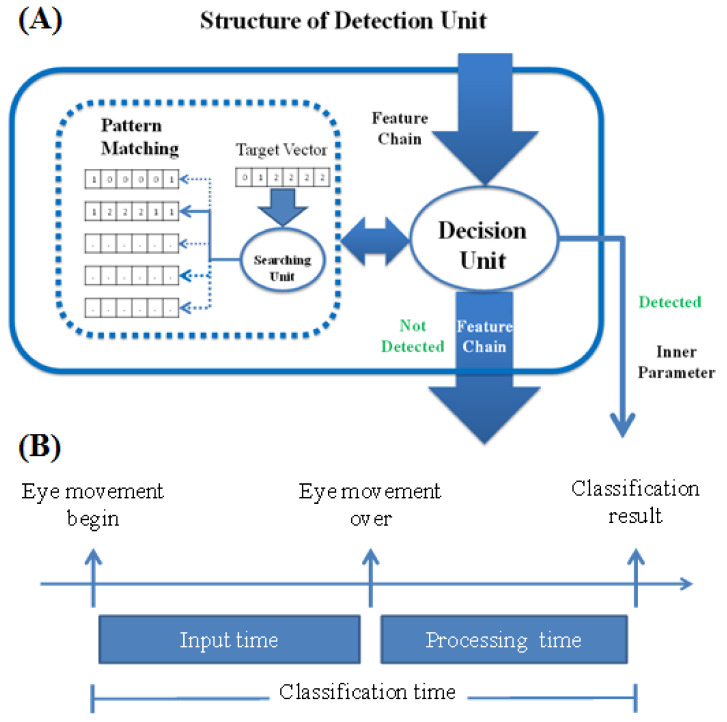
Detailed structure of the detection unit. (**A**) In each classification, the classifier removes seven adjoining feature digits from the feature chain. These digits form a seven-digit-long vector. The vector size is decided by both the sampling rate and the time duration of each type of eye movement. (**B**) Composition of the classification time. Because our study aims to design a real-time eye-movement detection system, the delay time between the actual eye movement and classification result feedback should be as short as possible. From the many experiments we conducted, we found that the eye-blink duration was the shortest among all the eye movements we studied. Thus, we take the eye-blink duration into consideration. Due to the characteristics of our classifier, we can use the symmetry of the EOG signals. This allows us to reduce the input time to half the eye-blink duration. In general, the duration of one blink is approximately 0.3 s. Thus, we set the input time to 0.15 s. Once the input time is decided, the size of the input feature vector is also determined.

In each classification, the classifier removes seven adjoining feature digits from the feature chain. These digits form a seven-digit-long vector. The vector size is decided both by the sampling rate and the time duration of each type of eye movement (Figure 8A).

Because our study aims to design a real-time eye-movement detection system, the delay time between actual eye movement and classification result feedback should be as short as possible. From the many experiments we performed, we found that the eye-blink duration was the shortest among all the eye movements of interest in this study (Figure 4B). Thus, we take eye-blink duration into consideration. Due to the characteristics of our classifier, we can use the symmetry of EOG signals. This allows us to reduce the input time to half the eye-blink duration. In general, the eye-blink duration is approximately 0.3 s. Thus, we set the input time to 0.15 s. Once the input time is decided, the size of the input feature vector Equation (13) is also determined (Figure 8B).

(13) Size of feature vector k=input time×sampling rate 

In this study, the input time is 0.15 s, and the sampling rate is 51.2 Hz. Thus, according to the above equation, the size of the feature vector is 7.68; we choose 7 to be the size of the vector.

### 2.4. Human–Computer Interface

**Figure 9:**
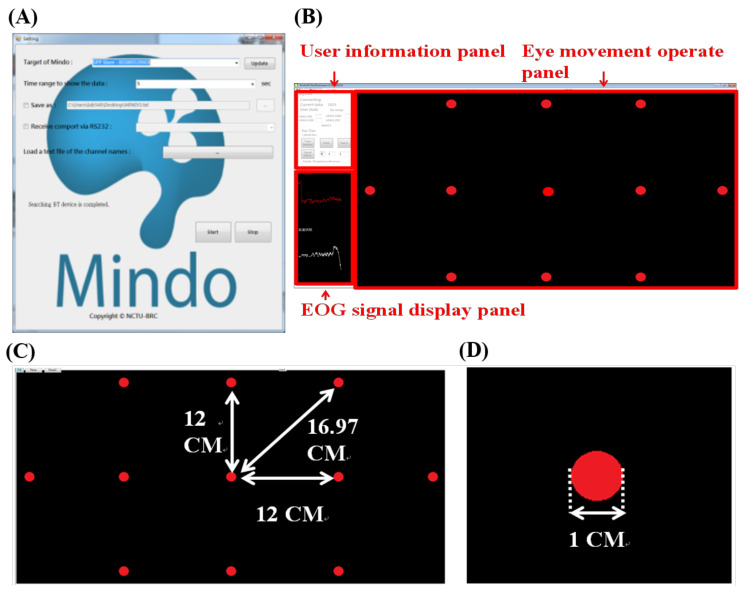
Configuration panel and normal mode of the proposed HCI system. (**A**) Configuration panel of the proposed HCI system. (**B**) Normal mode of the proposed HCI system. (**C**,**D**) Full-screen display of the normal mode.

Once the classification algorithm becomes sufficiently accurate and sophisticated, we utilize it to build an HCI system and implement it on a PC. In this section, the proposed HCI program is demonstrated and introduced. Figure 9A,B show the configurations of the panel. The user can configure the settings of the proposed system, such as the target board code, data display time range, and target destination of the record data. Once the settings are configured, the user can press the start button, and the program will execute in normal mode.

In the normal mode, the system distinguishes the eye movement performed by the user and outputs the classification result on the screen in real-time. The waveforms of the horizontal and vertical EOG signals are also shown in the EOG display panel (Figure 9B). In addition, the program can be executed in full-screen mode once users press the full-screen button in the upper-left corner (Figure 9C). In this research, we conduct an experiment to test the accuracy in full-screen mode, the detailed layout of which is shown in Figure 9C,D.

**Figure 10:**
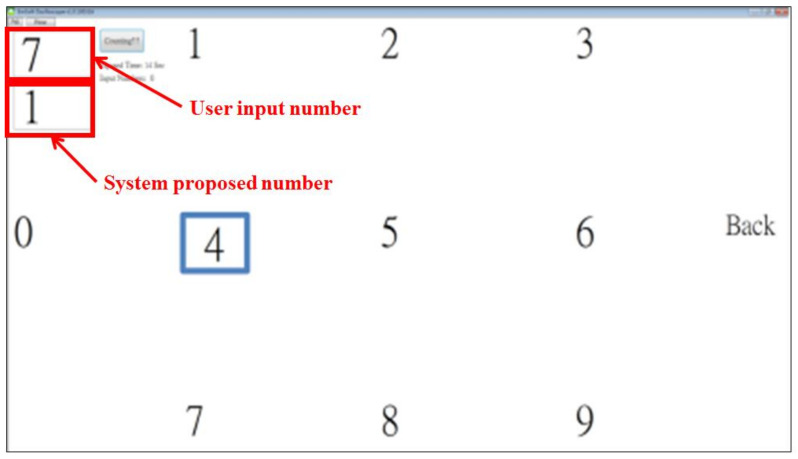
The developed phone-dialing mode. The user can use eye movements to control the target frame (look up, look down, look right and look left). For example, if the user moves his viewpoint from number five to number eight, the HCI system detects an up saccade movement and thus moves the target frame from number five to number eight. Once the target is on the target number that the user wants to choose, the user can simply double blink to input the target number. If an error occurs, the user can choose to go back and delete the incorrect number.

Figure 10 presents the phone-dialing mode, in which the user can use eye movements to control the target frame (look up, look down, look right and look left). For example, if the user moves his viewpoint from number five to number eight, the HCI system will detect an upward saccade and thus move the target frame from number five to number eight. Once the target frame reaches the target number that the user wants to choose, the user can simply double blink to input the target number. If an error occurs, the user can choose to go back and delete the incorrect number.

### 2.5. Experimental Goals

Our target is to design a sophisticated eye-movement detection system with HCI function in this study. The system should detect the current state of the eyes of a user in a robust manner. Thus, we focus on common eye activity, including ten different types of saccade, blink, and fixation movements. Additionally, we facilitate communicating with the computer for users performing these movements. For the above reasons, the following actions are taken to certify and assess the performance of our system: (1) inspection of the classification accuracy [36] of different eye-movement tasks from different users; (2) verification of the function of this system at the HCI level; and (3) justification of the practicality of the HCI system combined with a dry sensor.

**Figure 11:**
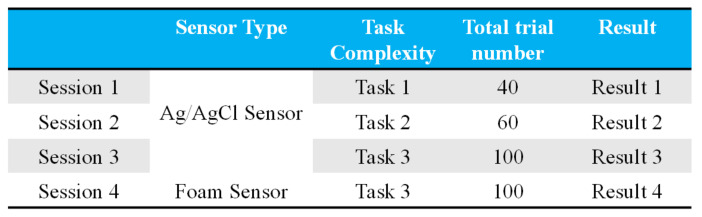
Details of experiment 1. In these experimental sessions, we focus mainly on the classification rate in the detection of different saccades for different tasks and sensors.

**Figure 12:**
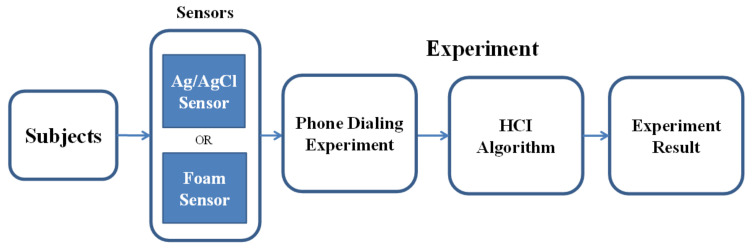
Diagram of the analysis method of the HCI performance test. In Experiment 2, we turn our focus to the performance of the HCI system in the eye-dialing phone number task.

Thus, according to these indicators, we design two experiments to reveal our system’s practicality, stability, and performance. Experiment 1: As depicted in Figure 11, we focus mainly on the classification rate of saccade detection with different tasks and sensors in this experimental session. Experiment 2: As depicted in Figure 12, in this experimental session, we focus on the HCI system’s performance when using the eye-dialing phone.

Because our aim is to detect different types of eye movements of users in daily life, the experimental environment should be as close to real life as possible. Thus, in the choice of the experimental apparatus, we select a common PC and monitor as our experimental operation platform. We designed the EOG signal input device, as previously mentioned. The experiments were conducted in a conventional laboratory during regular working hours. Participants were seated in front of a 22-inch flat-screen monitor at a distance of approximately 60 cm. The following experiments involved six healthy male subjects (20–28 years of age; mean age: 23.5 years) at National Chiao Tung University, Hsinchu, Taiwan. The experiments reported in this paper were approved by the Institutional Review Board (IRB) of National Chiao Tung University (NCTU-REC-106–057) and followed the rules of the Declaration of Helsinki. All subjects had normal or corrected-to-normal vision and normal auditory function. None of them reported any history of psychiatric disorders or neurological diseases. Additionally, people who consumed or used caffeine, tobacco, alcohol, and drugs were excluded from participating in the experiment. Before the experiment, every subject was asked to provide written informed consent and was presented with the safety rules displayed on the form.

### 2.6. Experimental Procedure

#### 2.6.1. Experiment 1

**Figure 13:**
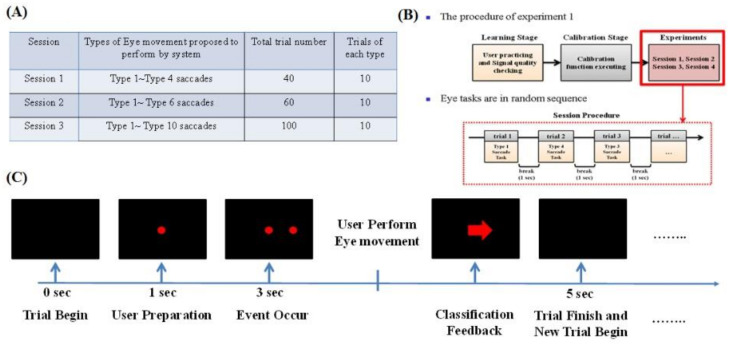
(**A**) Details of the sessions of experiment 1. (**B**) Detailed process of experiment 1. (**C**) Trial process of experiment 1.

In experiment 1, the system proposes that subjects perform different tasks in random order. EOG information is recorded and passed to the classifier during the experiment. Meanwhile, systems feed the classification result back to the monitor in real-time. The eye-movement tasks of each experiment are listed in Figure 13A. For example, in session 1, subjects are asked to perform Type 1 to Type 4 saccades. This means that users are only required to carry out saccades, down saccades, left saccades, and right saccades during session 1. The details of each movement type can be found in Figure 13A.

The process of the experimental trials is shown in Figure 13B,C. The screen is initially blank. Then, after 1 s, a red dot appears in the middle of the screen. The subject has to stare at this point. After 3 s, a new red point appears on the screen. The position of this new point is determined by the event type. The order of event types follows a random sequence. Both red dots disappear after 5 s, and then a new trial begins.

Once subjects perform the eye movements, the classification algorithm captures the occurrences and presents the classification result on the screen (Figure 13B). Thus, subjects can view the results corresponding to what they performed as classified by the system. In each trial, when an event occurred, we observed whether users performed any eye movements. Once eye movement occurs, the first classification result is presented after eye movement. The classification result is regarded as the tag of this trial. More details of the experimental results are discussed in the Results section.

#### 2.6.2. Experiment 2 (Phone-Dialing Experiment)

In experiment 2, two sessions are held with different types of sensors. As discussed in the previous section, people can use their eye movement to input a telephone number. The system generates ten numbers in a random sequence. Each subject inputs these ten numbers into the system by using their eyes. Once all ten numbers are input, the time spent inputting the ten numbers is recorded. This experiment verifies the efficiency of our eye-dialing system.

## 3. Results

### 3.1. Experiment 1

#### 3.1.1. Sessions Involving Four Types of Eye Movement with Ag/AgCl Electrodes

In this experiment, users are asked to perform four types of saccade movements (up, down, left, right), blinking and fixation. Table 1 shows the classification accuracy of session 1. In session 1, the best accuracy achieved was approximately 98% for subjects S2, S3 and S5. The accuracies of all subjects exceed 90%. In terms of movement type accuracy, the best accuracy occurs when the eye movement involves looking up. However, the accuracy when looking to the right is below 90%.

Table 2 is the summed confusion matrix for all participants normalized across the ground-truth rows. Correct classification is shown along the diagonal, and substitution errors are shown along the f-diagonal. The largest between-class substitution errors that occur are approximately 13%. Error occurs between the right-side movement direction and the null type. The best classification accuracy achieved was 100%, which occurred when looking up.

#### 3.1.2. Sessions Involving Six Types of Eye Movement with Ag/AgCl Electrodes

In this experiment, users perform six types of saccade movements (up, down, left, right, farther left, farther right), blinking and fixation. Table 3 shows the classification accuracy of experiment 1. The best accuracy achieved is 95%. However, unlike the previous session, the average classification accuracy decreases to only 88.3% for all subjects. The accuracies of all subjects exceed 80%. Moreover, in terms of movement type accuracy, the best accuracy is achieved for the up saccade, as in the previous session. Furthermore, in this session, the performances of left-side-related eye movements were slightly worse than those of right-side-related eye movements. This result is different from the previous experiment.

Table 4 is the summed confusion matrix for all participants normalized across the ground-truth rows. From the table, we can observe that between-class errors likely occurred between the looking-up action and other movement types. The largest between-class substitution error is approximately 11.7%, which occurred between the right-saccade and up-saccade movements. The best classification achieved was 93.3%, which occurred for up-saccade classification.

#### 3.1.3. Sessions Involving Ten Types of Eye Movement with Ag/AgCl Electrodes

In this session, users perform 10 types of saccade movements (up, down, left, right, farther left, farther right, up-left, up-right, down-left, down-right), blinking and fixation. Table 5 shows the classification accuracy of session 3. In this session, the best result among all subjects was the 96% accuracy of S3. The total average classification rate is 87.67%, which is only lower than that in session 2. Moreover, as expected, the performance for all normal saccades (up, down, left, and right) exceeds 90%.

Table 6 is the summed confusion matrix for all participants normalized across the ground-truth rows. From Table 6, we can observe that between-class errors likely occurred between up-saccade movement and other movement types. The largest between-class substitution error is approximately 13%, which occurred between the left saccade and up saccade. The best classification achieved was 96%, which occurred for the up saccade.

#### 3.1.4. Sessions Involving Ten Types of Eye Movement with Foam Sensors

In this final session of experiment 1, users perform 10 types of saccade movements (up, down, left, right, farther left, farther right, up-left, up-right, down-left, down-right), blinking and fixation with a foam sensor [34]. The signal-to-noise ratio of the developed system is approximately 91 db. Table 7 shows the classification accuracy results. In this session, the best result among all subjects was 93%. In addition, the average classification accuracy is 88.6%. Similar to the previous experimental session, we observe good performances for the up-, down-, left- and right-saccade movement classifications. Additionally, we observe that in this session, the performance of the down-right classification is considerably low, i.e., only 66%.

Table 8 is the summed confusion matrix for all participants normalized across the ground-truth rows. From the table, we can observe that between-class errors likely occurred between the up-saccade movement and other movement types. The largest between-class substitution error is approximately 32% according to this table, which occurred between the down-right saccades and right saccades. Additionally, as in the previous experimental session, there is a phenomenon in which the classification error is high between the up saccades and other saccades. We discuss this phenomenon further in the next section.

### 3.2. Experiment 2 (Phone-Dialing Experiment)

**Figure 14:**
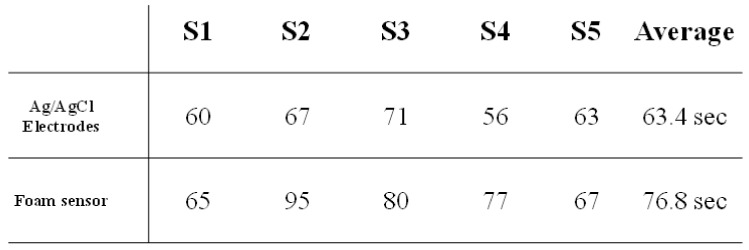
Results of experiment 2 (phone-dialing experiment). We find that the time spent under the Ag/AgCl electrodes is always less than that under the foam sensors. The best performance achieved is that of S4 when using the Ag/AgCl sensors. The average time spent when using the Ag/AgCl electrodes is 63.4 s, and that when using the foam sensors is 76.8 s. Finally, it should be noted that all the elapsed times are less than 90 s, except for those of subject 2.

In experiment 2, we test the performance of the proposed HCI system; the experimental results are presented in Figure 14. From Figure 14, we can see that the time spent by the Ag/AgCl electrode is less than that spent by the foam sensor. The best performance is achieved by S4 with the Ag/AgCl sensor. The average time spent by using the Ag/AgCl electrode is 63.4 s and that spent by using the foam sensor is 76.8 s. Finally, it should be noted that all the elapsed times are less than 90 s, except those of subject 2.

## 4. Discussion

In experiment 1, four sessions are conducted for each user. In experiment 2 (phone-dialing experiment), two sessions are conducted with different sensors. From the above two sets of experimental results, some information about our system can be extracted.

### 4.1. Oblique-Direction Error

From the results, it is apparent that the oblique-direction movement detection accuracies are typically lower than the normal-direction detection accuracies (i.e., up, down, left, and right). From the results, we find that when subjects perform oblique-direction eye-movement tasks, the system can mistakenly classify these movements as normal movements. For example, right-up movements have a 16% possibility of being classified as up movements. Right-down movements have a 10% possibility of being regarded as right movements and down movements. To determine the reason why between-class errors occur, we look at the original EOG signal data recorded during the experiments. We observe that the system faithfully transforms the input data into the correct result without any mistake. Additionally, when we check the experimental trial data in which the errors appear, we note that these between-class errors originate from mistakes made by the subjects. Specifically, while performing an oblique eye-movement task, some subjects could not carry out the correct eye movements. For instance, a right-up task was proposed, but subjects performed a right-right-up eye movement instead. These incorrect behaviors may be caused by personal eye use habits or fatigue. This is a significant topic that should be carefully treated.

### 4.2. High Classification Accuracy

As previously mentioned and discussed, studies involving biosignals always encounter intersubject variation. EOG is also confronted with this problem. This problem becomes more serious and unavoidable when realizing an EOG-based HCI system. From the experimental results, we note that all six users achieve classification accuracies above 80%. This result means that our classification is stable and successfully rejects the intervariation.

### 4.3. Robustness in the Vertical Direction

Due to its natural characteristics, the EMG signals sourced from blinks are inevitably collected by sensors when we measure EOG signals. Especially regarding vertical EOG signals, the EMG signals sourced from blinks and EOG signals generated by vertical eye movement are sometimes almost identical. Thus, in the eye-movement detection research field, it is important to determine the influence of blinking. From the experimental results in the previous section, we observe that the classification accuracy in vertical-direction eye movement approaches almost 100%. This shows that our system can resist the impact caused by blinking in vertical-direction EOG signals.

### 4.4. Error between up Saccades and Other Saccades

In the above experimental results, we find that the system algorithm is likely to classify other types of saccades as up saccades. This phenomenon seriously negatively impacts the performance of the algorithm. Thus, we reinspected the EOG raw data recorded during the experiment and reexamined the algorithm structure. Finally, we determined the reason for this phenomenon: the characteristics of blinking in the time domain are similar to those of up saccades. When a user was asked to perform the target movement by cueing the system, some unexpected blinks may have occurred at the same time. These blinks would be contained in the EOG signal and thus affect the performance of the classification algorithm. In this case, the system would distinguish these trials as up saccades.

### 4.5. High Classification Performance with Wearable Goggles and Foam Sensors

**Figure 15:**
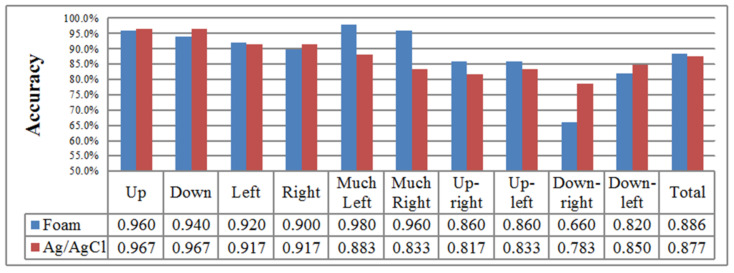
Comparison result between foam and Ag/AgCl sensors. This table presents the accuracy comparison between the Ag/AgCl and foam sensors. From this table, the accuracy of the down-right saccade for session 4 is fairly poor compared with the accuracy for session 3. This result occurs because when subjects were equipped with our wearable goggles, the dry sensors would be tightly attached to their faces. However, at the same time, these dry electrodes applied pressure to their skin. This pressure slightly limited their eye movements and thus negatively impacted the eye-movement performance.

From the experimental results of session 4 in experiment 1, we determine that the classification rate is considerably better than we expected. The overall average accuracy was 87.67% in session 3 and 88.6% in session 4. These results prove that our system is compatible with dry sensors. However, there are still some obstacles to overcome. Figure 15 shows the accuracy comparison plot between the Ag/AgCl sensor and the developed foam sensor [34]. From Figure 15, it is clear that the accuracy of the down-right-saccade classification is fairly poor for session 4 compared with that for session 3. This result occurs because when subjects were equipped with our wearable goggles, the dry sensors were tightly attached to the face. However, at the same time, these dry electrodes placed pressure on the skin. This pressure slightly limited eye movement and thus degraded the eye-movement detection performance.

### 4.6. The Practicality of the Proposed HCI System

From the result of experiment 2, we find that the time spent was always less than 90 s. This fact proves the suitability of the proposed HCI system for people with disabilities. Additionally, as we expected, the Ag/AgCl electrode required more time to be spent than did the foam sensor. This is because the stability of the proposed HCI system with a foam sensor is indeed less than that of the system with Ag/AgCl electrodes. This is an important issue to be resolved and improved.

Overall, the aim of this work is to create a wearable EOG-based system for ten-directional eye-movement detection, consisting of dry sensors/electrodes, a wireless acquisition device, and an eye-movement classification method to classify EOG signals. One of the advantages is using dry electrodes, which can be utilized with good signal quality and provide good electrical conductivity, resulting in the effective acquisition of EOG signals. Compared to other related papers/systems [37,38,39], users can reduce skin preparation using dry electrodes and achieve highly accurate EOG signals. In addition to the use of dry sensors, the developed acquisition device is lightweight and can be integrated into glasses. Currently, the total weight of the detection system (i.e., including the goggles and circuit boards) is approximately 150 g. Note that our long-term test results are available continuously for more than several hours. If Bluetooth low energy (BLE) is used, it is possible to use this system continuously for several days. In the future, there is an opportunity to embed eye movement recognition software into a smartphone app, use the smartphone screen or the smartphone to map to a large TV as the input medium, and through the smartphone or even the cloud platform or edge computing, realize input recognition, so there is no need to perform identification calculations on the PC.

Regarding the issue of motion artifacts, we observe this issue at the initial stage during the mechanism design, as mentioned. We try to avoid this problem through good fixation based on the outlook design. However, we may need to consider removing motion artifacts through artifact removal methods. Several artifact removal methods have been proposed to remove artifacts [40]. However, most of these methods cannot support real-time implementation or can reject only a few kinds of artifacts. Therefore, these methods are difficult to implement and apply to real applications. Recently, we developed an automatic artifact detection algorithm, including a novel artifact feature extraction method and a support vector machine (SVM) classifier [41]. As a result, the proposed method can effectively reject artifacts from biosignals and still maintain the phenomena of biosignal activities. In particular, our plans are to implement our recently developed automatic artifact detection algorithm [41] in this developed EOG-based system and then verify it on healthy subjects first. Along with new IRB approval for the inclusion of subjects with disabilities, the developed wireless HCI system has the potential to be used in daily life applications (including those of subjects with disabilities) in the future. At that stage, with actual data from subjects with disabilities, a reliable result (with statistical significance) for the developed system can be obtained.

## 5. Conclusions

In this study, an EOG-based HCI based on eye-movement detection is proposed to establish an alternative way to transmit information between humans and computers. This system can detect different eye movements, including fixation, blinking and ten different types of saccades (up, down, left, right, farther left, farther right, up-right, up-left, down-right and down-left), performed by users in real-time with high classification accuracy. In addition, the proposed system provides an eye-dialing interface that can be used by people with disabilities. Due to the design of the proposed EOG acquisition device, this system is lightweight, portable, wireless and comfortable to use. Compared with other eye-tracking methods and devices (for example, infrared oculography, video-based eye trackers and scleral search coils), the proposed system offers much practicality in real life. Additionally, a classification algorithm with a real-time function is designed for general purposes. The experimental results show that the best subject performance achieved, which occurred in session 3 under high complexity, was 96%. Additionally, the average classification rate of all subjects in session 3 was 87.67%. In addition, the eye dialysis experiments revealed the practicality of the proposed HCI system. Although our design attempts to provide a sophisticated system with stability and practicality for real-life use, there still exist some issues that should be addressed. As mentioned in the previous section, the variation problem has much impact on the classification rate. In our experimental session, as in other studies, we tried to control all the factors related to EOG signal measurement. However, for use in real life, a more robust algorithm that can reject the impact of extra movements, including head shaking, speaking, and walking, is needed to give the user a better experience. Additionally, with the development of computer intelligence research, many machine learning algorithms (ANN, SVM, fuzzy methods, etc.) have been proposed and have proven to be useful. Thus, it can be expected that our system performance would be improved by combining the classification algorithm with the above artificial intelligence methods. Finally, in addition to the above two issues, some topics of interest still exist, such as achieving a higher spatial resolution, reducing the classification time and achieving a more practical application. To help people with disabilities live independently and lead better lives, these issues urgently need to be overcome.

## Figures and Tables

**Table 1:** Experimental results of the 4-saccade-type eye task with Ag/AgCl electrodes. In this experiment, users were asked to perform four types of saccade movements (up, down, left, right), blinking and fixation. This table shows the classification accuracy of session 1. In session 1, the best result achieved was an accuracy of approximately 97.5% by subjects S2, S3 and S5. The accuracies of all subjects exceeded 90%. In terms of the movement type accuracy, the best accuracy was achieved for the looking-up eye-movement type. However, we can observe that the performance of the right saccade was sometimes below 90%.

	S1	S2	S3	S4	S5	S6	Total
Up	100%	100%	100%	100%	100%	100%	100%
Down	100%	100%	90%	90%	100%	100%	96.7%
Left	80%	100%	100%	80%	100%	100%	95.0%
Right	90%	90%	100%	70%	90%	90%	86.7%
**Total**	**92.50%**	**97.50%**	**97.50%**	**85.00%**	**97.50%**	**97.50%**	**94.6%**

**Table 2:** Summed confusion matrices of four types of eye tasks with Ag/AgCl electrodes. Correct classification is shown along the diagonal, and substitution errors are shown along the f-diagonal. The largest between-class substitution errors occur at approximately 13%. Error occurs between the right-saccade direction and null type. The best classification accuracy achieved is 100%, which occurred for the up-saccade classification.

		Classification Type				
		Up	Down	Left	Right	Unexpected
**Actual Type**	Up	**100%**	0%	0%	0%	0%
	Down	3.33%	**96.67%**	0%	0%	0%
	Left	1.67%	0%	**95%**	0%	3.33%
	Right	5%	0%	0%	**86.67%**	8.33%

**Table 3:** Experimental results of a 6-saccade-type eye task with Ag/AgCl electrodes. In this experiment, users perform six types of saccade movements (up, down, left, right, farther left, farther right), blinking and fixation. This table shows the classification accuracy of experiment 1. In this session, the best result achieved is 95%. However, different from the previous session, the average classification accuracy drops down to only 88.3% for all subjects. The accuracies of all subjects exceed 80%. In terms of the movement type accuracy, the best accuracy occurs for the up saccade, as in the previous session results. Moreover, in this session, the performances for the left-saccade eye movements are slightly worse than those of the right-saccade eye movements. This result is different from that of the previous experiment.

	S1	S2	S3	S4	S5	S6	Average
Up	100%	70%	100%	100%	100%	100%	93.3%
Down	80%	90%	70%	90%	50%	100%	85.0%
Left	100%	70%	70%	80%	100%	80%	88.3%
Right	80%	80%	80%	90%	90%	100%	88.3%
Farther Left	100%	100%	70%	70%	90%	90%	88.3%
Farther Right	90%	90%	90%	80%	70%	100%	86.7%
**Total**	**92%**	**83%**	**92%**	**85%**	**83%**	**95%**	**88.3%**

**Table 4:** Summed confusion matrices of six types of eye tasks with Ag/AgCl electrodes. From this table, we can observe that between-class errors are likely to occur between up saccades and other movement types. The largest between-class substitution error is approximately 11.7%, which occurs between the right saccade and up saccade. The best classification result achieved is 93.3%, which occurred for the up-saccade classification.

		Classification Type						
		Up	Down	Left	Right	Much Left	Much Right	Unexpected
**Actual Type**	**Up**	93.3%	6.7%	0.0%	0.0%	0.0%	0.0%	0.0%
	**Down**	10.0%	85.0%	0.0%	0.0%	0.0%	0.0%	5.0%
	**Left**	10.0%	1.7%	88.3%	0.0%	0.0%	0.0%	0.0%
	**Right**	11.7%	0.0%	0.0%	88.3%	0.0%	0.0%	0.0%
	**Much left**	3.3%	3.3%	5.0%	0.0%	88.3%	0.0%	0.0%
	**Much right**	11.7%	1.7%	0.0%	0.0%	0.0%	86.7%	0.0%

**Table 5:** Experimental results of a 10-saccade-type eye task with Ag/AgCl electrodes. Here, users perform 10 types of saccade movements (up, down, left, right, farther left, farther right, up-left, up-right, down-left, down-right), blinking and fixation. This table shows the classification accuracy of session 3. In this session, the best result among all subjects is that of S3, i.e., 96%. The total average classification rate is 87.67%, which is only slightly lower than the result of session 2. As expected, the performances for all normal saccades (up, down, left, and right) exceeded 90%.

	S1	S2	S3	S4	S5	S6	Total
Up	100%	100%	100%	100%	90%	90%	96.67%
Down	100%	100%	100%	100%	100%	80%	96.67%
Left	70%	90%	100%	90%	100%	100%	91.67%
Right	90%	100%	90%	100%	90%	80%	91.67%
Much Left	90%	70%	100%	80%	100%	90%	88.33%
Much Right	80%	40%	100%	90%	90%	100%	83.33%
Up-right	60%	90%	90%	80%	70%	100%	81.67%
Up-left	80%	90%	100%	70%	60%	100%	83.33%
Down-right	70%	100%	80%	50%	80%	90%	78.33%
Down-left	80%	100%	100%	70%	90%	70%	85%
**Total**	**82%**	**88%**	**96%**	**83%**	**87%**	**90%**	**87.67%**

**Table 6:** The correlation between brain dynamics and target detection behavior in the frontal region. We can observe that between-class errors likely occurred between the up saccades and other movement types. The largest between-class substitution error is approximately 13%, which occurred between the left saccades and up saccades. The best classification achieved is 93%, which occurred for the up-saccade classification.

		Classification Type									
		Up	Down	Left	Right	Much Left	Much Right	Up-Right	Up-Left	Down-Right	Down-Left
**Actual Type**	**Up**	96.67%	3.33%	0%	0%	0%	0%	0%	0%	0%	0%
	**Down**	3.33%	96.67%	0.0%	0%	0%	0%	0%	0%	0%	0%
	**Left**	6.67%	1.67%	91.67%	0%	0%	0%	0%	0%	0%	0%
	**Right**	3.33%	0%	1.67%	91.67%	0%	3.33%	0%	0%	0%	0%
	**Much left**	3.33%	0%	8.33%	0%	88.33%	0%	0%	0%	0%	0%
	**Much right**	1.67%	0%	0%	13.33%	0%	83.33%	0%	0%	0%	1.67%
	**Up-right**	13.33%	1.67%	0%	1.67%	0%	1.67%	81.67%	0%	0%	0%
	**Up-left**	16.67%	0%	0%	0%	0%	0%	0%	83.33%	0%	0%
	**Down-right**	1.67%	8.33%	0%	10%	0%	0%	0%	0%	78.33%	1.67%
	**Down-left**	3.33%	6.67%	5%	0%	0%	0%	0%	0%	0%	85%

**Table 7:** Experimental results of the classification accuracy for the 10-saccade-type eye task with foam sensors. The best result among all subjects is 93%. The average classification accuracy is 88.6%. Similar to the previous experimental session, we observe good performance in the up, down, left- and right-saccade movement classification tasks. Additionally, we observe that in this session, the performance of downright classification is considerably low, i.e., only 66%.

	S1	S2	S3	S4	S5	Total
Up	90%	100%	100%	100%	90%	96.00%
Down	100%	100%	90%	90%	90%	94.00%
Left	80%	100%	90%	100%	90%	92.00%
Right	90%	100%	80%	90%	90%	90.00%
Much Left	100%	100%	90%	100%	100%	98.00%
Much Right	100%	90%	90%	100%	100%	96.00%
Up-right	100%	60%	80%	100%	90%	86.00%
Up-left	100%	90%	70%	100%	70%	86.00%
Down-right	90%	80%	60%	40%	60%	66.00%
Down-left	80%	90%	80%	70%	90%	82.00%
**Total**	**93%**	**91%**	**83%**	**89%**	**87%**	**88.60%**

**Table 8:** Summed confusion matrices of 10 types of eye tasks with foam sensors. We can observe that between-class errors likely occurred between the up saccades and other movement types. From this table, the largest between-class substitution error is approximately 32%, which occurred between the down-right saccades and right saccades. Additionally, as in the previous experimental session, there exists a phenomenon in which the classification error is high between the up saccades and other saccades.

		Classification Type									
		Up	Down	Left	Right	Much Left	Much Right	Up-Right	Up-Left	Down-Right	Down-Left
**Actual Type**	**Up**	96%	2%	2%	0%	0%	0%	0%	0%	0%	0%
	**Down**	4%	94%	0%	0%	2%	0%	0%	0%	0%	0%
	**Left**	2%	2%	92%	0%	2%	0%	0%	0%	0%	2%
	**Right**	4%	4%	0%	90%	2%	0%	0%	0%	0%	0%
	**Much left**	2%	0%	0%	0%	98%	0%	0%	0%	0%	0%
	**Much right**	4%	0%	0%	0%	0%	96%	0%	0%	0%	0%
	**Up-right**	6%	0%	0%	8%	0%	0%	86%	0%	0%	0%
	**Up-left**	6%	0%	6%	0%	2%	0%	0%	86%	0%	0%
	**Down-right**	0%	2%	0%	32%	0%	0%	0%	0%	66%	0%
	**Down-left**	4%	0%	12%	0%	2%	0%	0%	0%	0%	82%

## Data Availability

Data will be provided on request through the corresponding author (L.-D.L.) of this article.
